# A concept of eliminating nonhomologous recombination for scalable and safe AAV vector generation for human gene therapy

**DOI:** 10.1093/nar/gkt404

**Published:** 2013-05-15

**Authors:** Biao Dong, Andrea R. Moore, Jihong Dai, Sean Roberts, Kirk Chu, Philipp Kapranov, Bernard Moss, Weidong Xiao

**Affiliations:** ^1^Department of Microbiology and Immunology, Sol Sherry Thrombosis Research Center, Temple University, Philadelphia, PA 19140, USA, ^2^St. Laurent Institute, One Kendall Square, Cambridge, MA 02139, USA and ^3^Laboratory of Viral Diseases, National Institute of Allergy and Infectious Diseases, National Institutes of Health, Bethesda, MD 20814, USA

## Abstract

Scalable and efficient production of high-quality recombinant adeno-associated virus (rAAV) for gene therapy remains a challenge despite recent clinical successes. We developed a new strategy for scalable and efficient rAAV production by sequestering the AAV helper genes and the rAAV vector DNA in two different subcellular compartments, made possible by using cytoplasmic vaccinia virus as a carrier for the AAV helper genes. For the first time, the contamination of replication-competent AAV particles (rcAAV) can be completely eliminated in theory by avoiding ubiquitous nonhomologous recombination. Vector DNA can be integrated into the host genomes or delivered by a nuclear targeting vector such as adenovirus. In suspension HeLa cells, the achieved vector yield per cell is similar to that from traditional triple-plasmid transfection method. The rcAAV contamination was undetectable at the limit of our assay. Furthermore, this new concept can be used not only for production of rAAV, but also for other DNA vectors.

## INTRODUCTION

Adeno-associated virus (AAV) is a nonpathogenic parvovirus that has been developed as a gene therapy vector (1). The applications for recombinant adeno-associated virus (rAAV) vectors are broad including both genetic and acquired diseases such as infections and cancer. The rAAV vectors are constructed by replacing the viral coding region containing the *rep* and *cap* genes with a desired therapeutic gene expression cassette. The resulting vector constructs contain only the inverted terminal repeats (ITRs) and generally do not share any homology with the *rep* and the *cap* genes in a helper construct, which are typically co-transfected together to support viral replication and packaging (2,3). Wild-type–like AAV, i.e. replication-competent AAV particle (rcAAV), is generally an undesired contaminant, which may be detrimental to the patient and can abrogate long-term transgene expression (4,5). Nevertheless, the rcAAV particles do get generated during the vector production process (6,7). Because AAV is a defective single-stranded DNA virus, the processes of replication and packaging of viral DNA occur primarily in the nucleus of host cells. Not surprisingly, until now all current AAV production methods necessitate the AAV helper genes to be transcribed in the nucleolus (8). Given the high numbers of the AAV vector genomes due to amplification in the nucleus of the host cell and the physical proximity with the helper genomes, nonhomologous recombination of AAV *rep* and *cap* genes and ITRs becomes unavoidable, which leads to the formation of rcAAV particles.

Another main bottleneck restricting the use of AAV vector is the lack of reliable, scalable and cost-effective production methods. The mainstay method for producing AAV vector used in research as well as clinical trials is based on triple plasmid transfection (9). However, it suffers from high costs and labor requirements that eventually cannot be scaled for wide-spread use in clinics. Therefore, a number of additional alternatives were sought. For example, various cell line–based rAAV production systems were reported (10). Another approach is to use various carrier vectors for rAAV production. Reported carriers for AAV *rep* and *cap* genes include baculoviruses, herpes virus vectors and adenovirus (Ad) (11,12). Because AAV Rep protein can interfere with adenoviral replication, only baculoviruses and Herpes viruses have been successfully used for rAAV production by a limited number of groups. However, these scalable strategies are not widely used owing to a variety of inherent difficulties.

Clearly, an alternative production method is required if rAAV is to be widely used in basic research and clinic. Here we report a new concept for producing AAV vectors by sequestering the AAV helper genes and the rAAV vector DNA in two different subcellular compartments. Taking advantage of the unique property of vaccinia virus (VV), the maintenance of its 200 kb genome in the host cytoplasm without the need to get into the nucleus (13), allowed for developing of this virus as a carrier expressing all necessary helper genes for AAV production. This method not only improves rAAV production by eliminating the need for the highly expensive and labor-intensive transfection-based method but also avoids ubiquitous nonhomologous recombination events, which lead to rcAAV particle formation. The prevention of rcAAV formation via differential intracellular localization of the helper and vector is a novel concept in the field as opposed to previous efforts aimed at reducing harmful effects of the hitherto unavoidable rcAAV particles via controlling their replication, packaging or expression (14–16).

Here we show the proof that this concept is not only feasible, but also provides a better alternative to existing methods. The vector yield per cell is similar to that from traditional triple plasmid transfection in suspension HeLa S3 cells with high scalable potential and with no rcAAV contamination detectable. The helper carrier can be easily removed from the vector preparation, making it amenable to the existing purification methods for preparation of clinical-grade material. The rAAV vector DNA can be integrated into the genomes of the host cells or be delivered by a nuclear targeting vector such as an Ad vector. Overall, this production system offers great flexibility for scalable production of high-quality rAAV vector for human clinical trials.

## MATERIALS AND METHODS

### Cell lines

HEK293 and HeLa S3 cells were cultured in Dulbecco’s modified Eagle’s medium supplemented with 10% fetal bovine serum, 100 µg of penicillin/ml and 100 U of streptomycin/ml (Invitrogen, Carlsbad, CA). CV-1, BSC-1 and 16095 (a human fibroblast cell line from Coriell Institute) were cultured in Minimum Essential Media with 10% fetal bovine serum, 100 µg of penicillin/ml and 100 U of streptomycin/ml. All cells were maintained in a humidified 37°C incubator with 5% CO_2_. To make stable cell lines with rAAV genome, HEK293 cells were transfected with pscAAV-CB-EGFP and pCI-neo by Lipofectamine 2000 and selected by 600 μg/ml G418. Single-cell–derived cell lines were then evaluated by rAAV production using plasmid transfection in 96-well plate.

### Plasmid constructs

The plasmid pRB21was digested with SmaI and StuI (NEB, Ipswich, MA), and then dephosphorylated by calf intestinal alkaline phosphatase (NEB, Ipswich, MA). This digested plasmid was used as the backbone to construct all plasmids having individual AAV2 *rep* and *cap* genes. AAV *rep78* and *rep52*, and AAV2 *vp1*, *vp2*, *vp3* genes were polymerase chain reaction (PCR) amplified by using plasmid pH22 as the template. To make the plasmids for recombinant VV having single *rep* or *cap* gene, individual PCR product was digested by EcoRV and blunt-end ligated into pRB21.

### Recombinant VV generation

To generate VVs, CV-1 cells were infected by vRB12 at multiplicity of infection (MOI) of 1 for 1 h followed by calcium phosphate transfection of pRB21-derived plasmids (17). Each kind of VV has undergone three rounds of plaque purification and then propagated in BSC-1 once. The resulting VV prep was used to prepare the virus stock in suspended HeLa S3 cells.

### Recombinant Ad production

Ad-AAV-CMV-EGFP hybrid vector was purchased from Penn Vector Core (Philadelphia, PA). Ad-AAV-CMV-LacZ was created using Ad-Easy kit (Strategene, La Jolla, CA). Briefly, pShuttle plasmid was digested by EcoRI, filled in by T4 DNA polymerase and dephosphorylated by calf phosphatase CIP. The CMV-lacZ expression cassette with two ITRs was excised from the plasmid pAAV-CMV-LacZ by PstI, and filled in by T4 DNA polymerase for blunt-end ligation. Ad-AAV-CMV-LacZ was then rescued according to the manufacturer’s instruction. The propagation and purification of Ad-AAV hybrids were carried out as previously described.

### Recombinant AAV vector production

rAAV vectors were produced by either triple plasmid transfection or using vaccinia helper carriers. Vector production and purification using triple plamsid transfection method has been described previously (18). To produce rAAV using vaccinia carrier and Ad-AAV hybrid infection in small scale, the host cells were then infected by VVs at MOI of 1 at 16 h after Ad infection. At 36 h after infection or VVs, the cells were collected. The vectors were released by three rounds of freeze/thaw. Residual Ad was heat inactivated at 56°C for 1 h for direct assay.

To produce rAAV vector in suspension cells, HeLa S3 cells were grown in 400 ml of growth media in a 1-liter Celstir* Spinner Double Side Arm Flask (Wheaton Science Products, Millville, NJ). When the cell density reached 2.4 × 10^6^ cells/ml, Ad-AAV-CMV-EGFP was added at MOI of 5. The cells were then infected with vv-w5 and vv-w8 at MOI of 1. The cells were collected at 36 h after infection. AAV vectors were then purified by two rounds of cesium chloride (CsCl)–gradient ultracentrifugation. After extensive buffer exchange against phosphate-buffered saline with 5% d-sorbitol, the purified virus was stored at −80°C before administration. rAAV-CMV-lacZ were produced similarly using Ad-AAV-CMV-lacZ with Ad5 (MOI 5) added as an enhancer. Vector purity and genome titer were analyzed by silver staining and Southern blotting. For AAV2-GFP or lacZ vector used in this study, each GFP or LacZ positive unit in 16 095 cells was correlated to 1 × 10^3^ vector particles determined by Southern blot or Silvering staining.

### Silver staining

Purified virus (∼1 × 10^10^ particles) was mixed with protein loading dye and heated at 95°C for 5 min. The denatured samples were then separated on 10% sodium dodecyl sulphate–polyacrylamide gel electrophoresis (SDS-PAGE) gel. After electrophoresis, the gel was stained by Pierce® Silver Stain Kit (Thermo Scientific, Rockford, IL) according to the manufacturer’s instruction.

### Western blot hybridization

Total proteins were extracted with lysis buffer, which consisted of 50 mM Tris at pH 8.0, 150 mM NaCl, 1% Triton X-100, 10 mM DTT and 1× protein inhibitor (Roche, Indianapolis, IN). Cell lysates were resolved on 10% SDS-PAGE gel and transferred to nitrocellulose membrane (Bio-Rad, Hercules, California). After blocking the membrane with 5% nonfat dry milk in TBST buffer, which contains 25 mM Tris·HCl at pH 8.0, 150 mM NaCl and 0.1% Tween 20, the membrane was incubated with the primary antibody, anti-AAV capsid (B1, American Research Products, Belmont, MA) or anti-AAV Rep (303.9, American Research Products), at a dilution of 1:500 at 4°C overnight. The membrane was washed and incubated with a horseradish peroxidase (HRP)-conjugated sheep anti-mouse IgG antibody (Sigma, St Louis, MI) at a dilution of 1:2000. The membrane was developed using an enhanced chemiluminescent substrate (Amersham-Pharmacia Biotech, Piscataway, NJ).

### Detection of rcAAV

rcAAV was sequentially amplified and detected as described previously (6). For the first amplification, HEK293 cells were grown in six-well plates overnight to a confluence of 70–80%. Wild-type Ad Ad5 was added to each well at MOI of 5, and AAV vectors were added at various genome particles, which is from 10^7^ to 10^12^. The cells were collected at 72 h after infection, and subjected to three cycles of freeze/thaw, followed by being heated at 56°C for 1 h. For the second amplification, HEK293 cells were infected with Ad5 and the above freeze/thaw lysates for 72 h. Cells were then pelleted and washed once by PBS. Hirt DNA was extracted and dissolved in 30 μl TE (10:1). PCR was carried out in a 25 μl of system with 35 cycles of 1 min at 94°C, 1 min at 55°C and 1 min at 72°C.

### Animal procedures

The 4–5-week-old C57BL/6 mice were housed in a pathogen-free environment on a normal diet. All surgical procedures were in accordance with the institutional guidelines under approved protocols at Temple University. Three hind limb muscles were injected independently with 5 × 10^10^ purified viral particles. After 9-week injection, the mice were sacrificed and the target muscles were frozen immediately in isopentane, which was chilled in liquid nitrogen. The muscles were then sectioned at 10 μM in thickness. The EGFP signals from the frozen sections of muscle samples were examined under Leica DM1 3000 B. For hemophilia A–related work, the procedures have been described previously (18).

## RESULTS

### The concept of cytoplasmic helper vector for rAAV production

**Figure 1.**
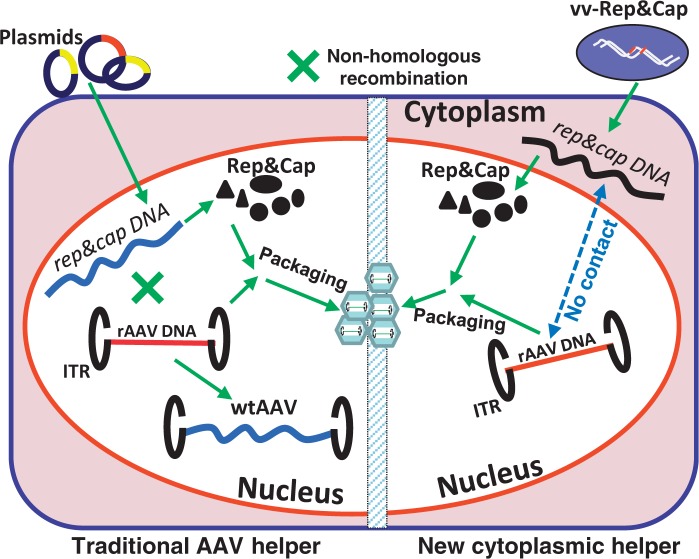
Schematic representation of the concept of eliminating replication competent wild-type–like AAV particles from a rAAV production system. Major components of the system proposed in this work and the traditional system are illustrated. The sequestration of the carrier DNA (‘*rep*&*cap*’) and rAAV-DNA into two different cellular compartments eliminates the physical contact of *rep&cap* and rAAV DNA and therefore the possibility of generating wild type (wtAAV), i.e. rcAAV particles. The ‘*rep&cap*’ denotes the corresponding genes and ‘Rep&Cap’ - proteins.

All of the existing methods of rAAV production suffer from known shortcomings that limit their wide-scale application in basic research and clinic. The most commonly used method based on transfection suffers from high cost in money and labor—for example, its took 432 independent 10-stack culture chambers to produce enough vector for a phase I hemophilia B clinical trial, clearly not scalable for large animal model experiments or broad clinical application (9). One common thread that is shared by all current vector production systems is that they are all based on the same intuitive thinking that the *trans* and *cis* elements required for packaging of AAV, which takes place in the nucleolus (1,8,19), must also reside in the nucleus of the host cells. Here we present a new concept that breaks from this paradigm. It is based on sequestering the AAV *rep* and *cap* genes and the AAV vector DNA in two separate compartments of the host cells as illustrated in Figure 1. The implementation of this strategy requires a carrier to confine the helper DNA to the cytoplasm. Besides RNA viruses, the only DNA viruses that have this capability are members of the poxvirus family, such as VV, which are well known for carrying out their entire life cycle within the host cytoplasm. Moreover, VV has been developed as a protein expression vector that found wide application in human vaccines and is able to direct high-level transgene expression in the target cells. As shown in Figure 1, we envisioned a scenario, whereas the genomic DNA of the helper virus containing the *rep* and the *cap* gene resides in the cytoplasm, is transcribed and translated there and the resulting Rep and Cap proteins migrate into the nucleus to ensure rAAV vector replication and packaging. The rAAV vector DNA can be incorporated into the host chromosome or delivered to the nucleus by an Ad-AAV hybrid. In either strategy, there will be no transfection procedure necessary and the rare Rep protein mediated or nonhomologous recombination events between the helper DNA and vector DNA can be eliminated systemically in the vector production system (7,16,20,21). In theory, this strategy could be applied to produce multiple DNA vectors including but not limited to Ad vectors. Here, we adapt it to rAAV vector production as a proof of this concept.

>

### Optimization of carrier vectors for rAAV vector production

**Figure 2.**
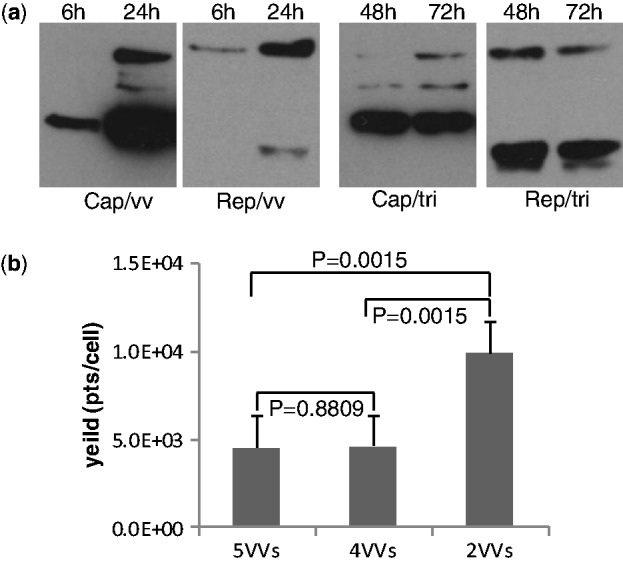
Vaccinia helper carrier-supported AAV helper gene expression and rAAV production. (**a**) Rep and Cap expression profile of vaccinia carrier: panels marked ‘Cap/vv’ and ‘Rep/vv’, respectively. VVs (vv-Rep78, vv-Rep52, vv-VP1, vv-VP2 and vv-VP3) were used to infect HeLa S3 cells at MOI of 1 along with Ad-AAV-CMV-EGFP infection (MOI 5) at 16 h earlier. The cells were collected at the time identified, and the expression of *rep* and *cap* was analyzed by western blot. The expression of *rep* and *cap* in the triple plasmid method is also shown: panels marked ‘Cap/tri’ and ‘Rep/tri’, respectively. (**b**) Vector yield comparison using different combinations of vaccinia carriers. HeLa S3 cells were infected by Ad-AAV-CMV-EGFP at MOI 5 followed by infection of 5VVs (vv-Rep78, vv-Rep52, vv-VP1, vv-VP2 and vv-VP3), 4VVs (vv-Rep78, vv-Rep52, vv-VP1 and vv-VP2) or 2VVs (vv-w5 and vv-w8). AAV-CMV-GFP vectors were harvested 36 h later. After heat inactivation, cell lysate derived from 1 × 10^4^ cells was used to infect 5 × 10^5^ 16 095 cells. The y-axis shows the yield measured by flow cytometry. Each GFP positive cell represents 1 × 10^3^ AAV viral particles.

VV retains its DNA genome in the cytoplasm of the host cells. It possesses unique transcription control elements, which allow its DNA to be transcribed in the cytoplasm. In contrast to RNA produced in the nucleus, RNA synthesized from VV genomes is not subjected to RNA splicing due to the lack of splicing factors in the cytoplasm. AAV has a small single-stranded DNA genome of only ∼4700 nucleotides. The proteins needed for AAV replication and packaging (Rep78, Rep52, VP1, VP2 and VP3) are encoded in two overlapping reading frames, which are normally translated from spliced mRNA. Earlier studies have demonstrated AAV Rep proteins expressed from VV were functional (22). Therefore, each AAV protein needs to be expressed independently by vaccinia vectors. Because Rep68 and Rep40 can be omitted in the AAV production system (23), we constructed the vaccinia vectors vv-Rep78, vv-Rep52, vv-VP1, vv-VP2 and vv-VP3 to express the corresponding proteins. As expected, vv-Rep78 and vv-Rep52 expressed the desired gene products (Figure 2a). Interestingly, VP2 and VP3 could be expressed from a single promoter in vaccinia vector vv-VP2 (data not shown), and this also confirmed earlier studies that minor spliced RNA expresses VP1 and major spliced RNA expresses both VP2 and VP3 (24–26). As shown in Figure 2a, a co-infection of all vaccinia carriers at equal ratio resulted in a pattern where VP1 and VP3 are overrepresented compared with VP2, which contrasts with what is observed with the commonly used triple plasmid transfection system. Expression of Rep78 from the vaccinia carrier was found to be higher than that of Rep52, which was an added benefit of this system because robust Rep78 expression appeared to be essential for high-yield vector production due to shorter incubation time after vaccinia infection. In contrast, the optimized helper plasmid pH22, routinely used by us in the transfection protocol, was engineered to have lower levels of Rep78 levels relative to Rep52 to increase rAAV titer due to longer incubation hours (72–96 h) (27).

>

To confirm that the proteins expressed by the vaccinia carriers can support rAAV production, we attempted to use all five vaccinia vectors along with the transfected AAV vector plasmids and mini-Ad plasmids in HEK293 cells (summarized in Figure 2b). As expected, infectious rAAV with a GFP reporter can be produced. As the next step, we attempted to reduce the number of vaccinia carriers necessary for AAV production. We were able to reduce the required vaccinia carriers to four instead of the original five: vv-Rep78, vv-Rep52, vv-VP1 and vv-VP2 were shown to be sufficient for rAAV production without reduction in vector yield. We then combined Rep78 and VP2 in one vaccinia vector vv-w5 and Rep52 and VP1 in vv-w8. The use of these two vaccinia carriers further improved the rAAV yield (Figure 2b), presumably due to a greater probability that each host cell was infected with two viruses compared with four as well as reduced cytotoxicity to the host cells resulting from the lowered MOI.

### Vaccinia carrier supports rAAV vector production in HEK293 cells with integrated rAAV genomes

**Figure 3.**
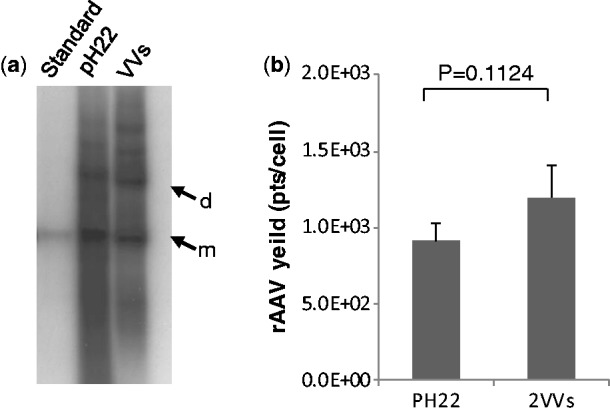
Vacccinia carriers support rAAV production in HEK293 cells with integrated rAAV genomes. The HEK293 cells with integrated pscAAV-CB-EGFP were selected for testing the vaccinia carrier helper function. The cells were infected with Ad-T7 (MOI of 5) for 6 h before infection by vv-w5 and vv-w8, at MOI of 1. In parallel, the cells were also transfected with pH22 + pfΔ6. The infected cells were harvested at 24 h after infection, whereas the transfected cells were collected at 48 h after transfection. (**a**) Profile of rAAV DNA replication in AAV cell line induced by vaccinia carrier infection (lane ‘VV’) or transfected AAV helper plasmids (lane ‘pH22’). Dpn I-digested Hirt DNA (10 µg) was used in each lane for Southern blotting with GFP-specific probe. m, monomer; d, dimer. The first lane is monomer-sized pscAAV-CB-EGFP as a standard (10 ng). (**b**) Comparison of rAAV yield using plasmid transfection or vaccinia helper carrier. Cell lysates derived from 10^4^ cells were used to infect 5 × 10^5^ 16 095 cells. EGFP positive cells were measured by flow cytometry. Each GFP positive cell represents 1 × 10^3^ AAV viral particles.

As mentioned above, a highly attractive feature for any new rAAV production system is the ability to avoid unscalable plasmid transfection procedures altogether. To demonstrate the advantages of using vaccinia helper carriers with AAV *rep* and *cap* genes, we generated a 293 cell line with integrated pscAAV-CB-EGFP, which carries a self-complementary AAV vector with a GFP reporter gene. We compared the rescue, replication and packaging of scAAV-CB-EGFP vectors using vaccinia carriers vv-w5 and vv-w8 or transfected AAV helper plasmid (pH22) along with mini-Ad plasmid DNA (27). These results are presented in Figure 3. The Southern blot analysis revealed that Rep proteins expressed from vaccinia carriers functioned properly in rescuing and replicating AAV vector DNA sequences. This is consistent with a previous study that Rep proteins expressed from a vaccinia vector were functional (22). The replication efficiency is also similar to that conferred by transfection with the conventional AAV helper plasmids as judged by the presence of similar level and pattern of viral DNA (both monomers and dimers, Figure 3a). The resulting vector yield per cell is slightly better than that obtained after plasmid transfection in terms of yield (Figure 3b).

>

### Scalable production of rAAV vectors with Ad-AAV hybrid and vaccinia carrier vectors

**Figure 4.**
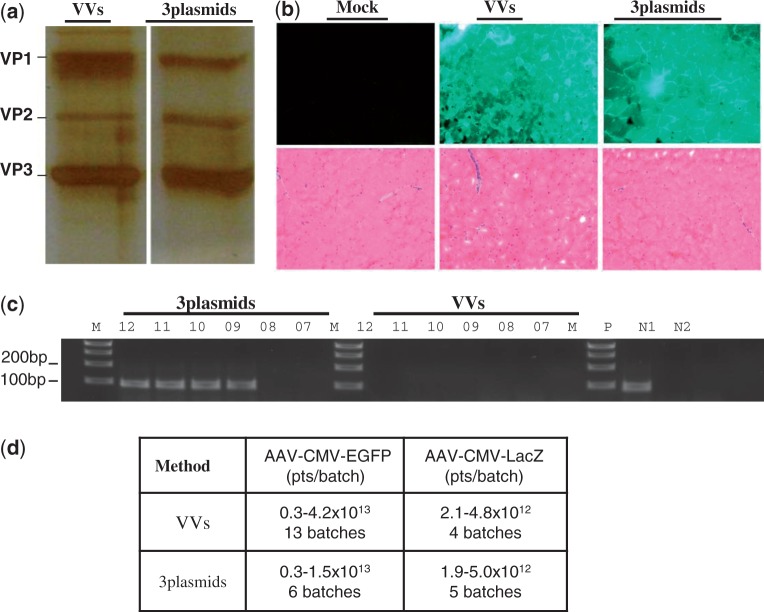
Scalable production of AAV vectors using vaccinia carrier and Ad/AAV hybrid in suspension cells. The suspension HeLa S3 cells were infected by Ad-AAV-CMV-EGFP and VVs (vv-Rep78, 52, vv-VP1 and vv-VP2). The cells were harvested at 36 h after infection and the rAAV vectors were purified by two rounds of CsCl gradient ultracentrifugation. (**a**) Silver staining of rAAV vector produced by vaccinia carriers or triple transfection methods (‘3plasmids’). Each lane includes 1 × 10^10^ purified AAV particles (pts) resolved on 10% SDS-PAGE. (**b**) AAV-CMV-GFP transduction of mouse muscle. C57BL/6 mice were injected with 5 × 10^10^ purified viral pts produced by the above procedure. The tissue was harvested at 9 weeks after injection. Sections (10 µm in thickness) were subjected to HE staining (lower) or direct GFP signal visualization under fluorescent microscope. (**c**) Detection of rcAAV after sequential amplification in HEK293 cells. Hirt DNA was extracted and used as the template for PCR (35 cycles) with primers specific for AAV coding region and separated on agarose gel. Vectors produced by triple plasmid transfection method (‘3plasmids’) and vaccinia carriers (‘VVs’) were analyzed. ‘P’, PCR positive control using plasmid template; N1, infection with Ad only; N2, infection with 1 × 10^12^ rAAV vectors only. The numbers on top of the gel lanes indicate the amount of vectors in logs being used for initial infection of HEK293 cells. (**d**) Summary of rAAV vector yield by the vaccinia helper (4VVs and 2VVs) as compared with triple plasmid transfection. The vector yield ranges and number of batches attempted are shown.

We then investigated whether it was possible to produce rAAV vectors on a large scale in suspension Hela S3 cells with vaccinia helper carrier. Because Ad-AAV hybrid can readily be amplified in large quantity and the performance is independent of integration loci in the host chromosome, it provides a preferred strategy for delivering AAV-CMV-EGFP vector DNA sequence into the nucleus as opposed to a cell line with integrated copy. In the particular example shown in Figure 4a–c, the vector yield of AAV2-CMV-EGFP was 1.5 × 10^13^ genomes from 400 ml suspension HeLa S3 cells. After purification by two rounds of CsCl gradient ultracentrifugation, the vector preps were further filtered through 0.22 µm filters to eliminate residual vaccinia carrier particles. Plaque assay showed that no vaccinia carrier could be detected in 1 × 10^12^ rAAV particles, suggesting the ultracentrifugation and filtration could completely remove the vaccinia carrier particles (data not shown). We then analyzed the rAAV capsid composition by silver staining (Figure 4a). It showed that the relative ratio of VP1, VP2 and VP3 of AAV2-CMV-EGFP produced by the new system was similar to that by triple plasmid transfection. This is in contrast to the differences in VP1, VP2 and VP3 ratio in the Figure 2.

>

The contaminating levels of rcAAV particles are shown in Figure 4c. After two rounds of recycling infection in HEK293 cells, rcAAV positive signal was detectable in 1 × 10^9^ genome particles in vector prepared by the triple plasmid transfection method. In contrast, no rcAAV particles could be detected in 1 × 10^12^ particles derived from the new method. To confirm the vector performance *in vivo*, the vector produced by the new method was then injected into the muscle of C57BL6 mice and resulted in strong GFP expression as shown in Figure 4b. The HE staining of mouse muscles also revealed no inflammation infiltration (Figure 4b). The resulting number of GFP positive cells indicates the efficiency with which the vectors can transduce the target tissue *in vivo*. Visual scoring of the infected tissues showed that the vectors from the new system works as efficiently as those from the traditional one as exemplified in Figure 4b.

Next, we attempted to produce multiple batches of Ad-AAV hybrid vectors containing either CMV-GFP or CMV-lacZ as reporter genes. The titers of these preparations ranged from 0.3 to 4.2 × 10^13^ particles, consistently comparable with that of triple plasmid transfection methods in terms of yield per cell (Figure 4d). These titers can be verified by multiple tittering methods including silver staining, dot blot and qPCR (Table 1). Vectors produced by both vaccinia carrier and plasmid helper after double CsCl gradient purification achieved similar potency of ∼1–2000 particles per transduction units (Table 1). Similar results were also obtained for the AAV-CMV-lacZ vector produced using the same methods (Figure 4d).

**Table 1.** Characterization of AAV vector produced by triple plasmid transfection and vaccinia carrier

Production methods	3plasmids	VV
Genome titer (qPCR, GC)	2.3 × 10^12^	2.1 × 10^12^
Genomic titer (Southern blot, GC)	5.6 × 10^12^	3.5 × 10^12^
Capsid titer (Silver staining, pts)	5.0 × 10^12^	2.5 × 10^12^
Transduction titer (flow cytometry, TU)	2.5 × 10^9^	2.3 × 10^9^
GC (Southern)/TU ratio	2.2 × 10^3^	1.5 × 10^3^
Ad helper DNA contamination (measured by qPCR)	0.034%	0.024%
AAV helper DNA contamination (measured by qPCR)	0.11%	0.0046%

AAV-CMV-EGFP vector was produced in HEK293 cells by triple plasmid transfection (3plasmids) or was produced in HeLa S3 cells infected with Ad-AAV hybrid virus and vaccinia helper viruses (VV). TU, transducing unit; GC, genome copy; Pts, particles.

## DISCUSSION

In this study, we have demonstrated a proof of a novel concept of production of DNA virus vectors based on sequestration of the helper and vector DNAs into respectively the cytoplasm and nucleus of a host cell. Theoretically, this should eliminate the previously unavoidable problem of nonhomologous DNA recombination between the genomes of the AAV vector and the helper (6,7). This is fundamentally superior to previous strategies that did not eliminate the wtAAV-like particles, but rather suppressed their packaging (7,14–16). The vector preparations produced by this new method resulted in no detectable levels of replication-competent recombination products at the maximum limit of our assay (none in 1 × 10^12^ particles), which is at least three orders of magnitude better than the results from the traditional method where the recombinants were detected among 1 × 10^9^ particles. Being free of the rcAAV contaminants should make this system a lot more attractive to the human clinical trials because no potential viral protein could be produced and complicate the therapeutic transgene expression directly or indirectly through hazardous immune responses.

Non-rcAAV form of DNA contaminants were documented to present an additional risk in human gene therapy (14). Although we did not address this issue directly, sequestering the capsid DNA sequence in the cytoplasm compartment would theoretically prevent co-localization of that DNA with the replicating vector genomes in the nucleus and thus might reduce such contaminants as well. This feature could be another potential benefit of the new system.

The vector yield per cell obtained by this method is comparable with the optimized conventional transfection methods (3,28). This suggests that the cytoplasmic vaccinia helpers have not adversely affected AAV replication and packaging. The conventional triple plasmid transfection method, despite its tedious procedures, high cost and inefficiency, generally yields satisfactory vector preparations in animal experiments and in preliminary human clinical trials (3,7,29). The vectors produced by this new method were equally efficient *in vivo* using GFP as a reporter gene (Figure 4b). Finally, using canine factor VIII as a secretable reporter, the same numbers of transduced viral particles made using the new and the standard procedures generated statistically indistinguishable levels of factor VIII in the plasma of the animals (Supplementary Figure S3). However, this new method would be scalable and cost-effective because the transfection procedure is no longer necessary: based on our preliminary results, 1 × 10^16^ rAAV vectors can be projected from just 100 liters of suspension Hela S3 cells. The unique advantages of rcAAV and scalable production should allow this new strategy to become the primary choice for rAAV vector production and thus replace the mainstream transfection-based system. Also, the method is flexible: vectors based on other AAV serotypes can be also made, only the vv-VP1 and vv-VP2 sequences need to be replaced in the carrier vectors so that the capsid proteins from the respective serotype are expressed. We have attempted production of vectors based on serotypes AAV8 and AAV9 and obtained similar results as with AAV2 (data not shown).

Although other scalable AAV vector production systems have been developed in the past decade (10,23,30,31), they have not seen broad adoption because of various issues. For example, the baculovirus production system alleviates the transfection problems but results in lower vector infectivity caused by production in insect cells as well as low yield for some vectors (32–36). Herpes vector is another option for carrying AAV helper function; however, there are difficulties in production of the carrier itself and concerns over contamination of the final preparations with rcAAV as well as with Herpes revertants (32,35–37). On the other hand, VV helper virus is stable and grows efficiently (Supplementary Figure S1 and Supplementary Table S1). Cell lines producing rAAV thus far have been inconsistent and unstable (38–40) and require extensive screening for functional ones to achieve maximum efficiency.

Any novel vector production system comes with concerns over stability of the helper carrier. In this respect, unlike Ad, VV growth is not inhibited by the AAV *rep* and *cap* gene products. The carrier helper can be grown easily (1 × 10^3^^−^^5^-fold amplification per each 24 h cycle) and after 10 consecutive passages, we did not detect any changes in AAV gene expression from vaccinia carriers (Supplementary Figure S1).

Thus, this entirely new rAAV production system avoids all of the above issues by eliminating rcAAV generation completely in theory while maintaining similar advantages of other vector production methods. Still, we envision room for further improvements in the vaccinia helper vectors beyond the current proof of concept study. Alternative VV strain such as modified vaccinia Ankara may be tested. VV is not a particular strong helper for AAV production. Essential Ad helper genes may be incorporated into vaccinia carrier as well. In this study, all AAV genes in the helper carrier vectors are under the control of the same promoter. Future optimization of the regulatory regions may further improve rAAV vector yield. Currently, we used two vaccinia carriers, however it may be possible to combine them into one vector because vaccinia carrier can accommodate >20–25 kb transgene sequences.

In terms of the mode of delivery of desired sequences to the nucleus, we argue that the Ad-AAV hybrid vectors have the advantage of short development time and the flexibility of MOI allowing precisely controlled initial gene copy number, an important factor for high rAAV yield. Importantly, performance of such vectors is independent of integration position in the host genome as opposed to the integrated rAAV DNA sequence. In addition, with the combination of vaccinia carrier and Ad-AAV hybrid, the rAAV vectors can be produced from many mammalian cells grown in suspension, not just HEK293 cells.

To prove the principle of the new method we have used CsCl centrifugation for rAAV purification. However, we do not see any conceptual hurdles to preparation of clinical-grade vector material by adopting the established standard operating procedures approved in the current clinical trials (41,42). The only difference in the immediate output material of this method and the other established methods is the presence of vaccinia carrier viruses at the time of harvesting. The latter, however, can be filtered with conventional 0.22 µm filters—capsid diameter of VV is 0.4 µm. Furthermore, the density of the VV is 1.24–1.27 g/cm^3^, which is significantly lighter than AAV (1.41 g/cm^3^). Thus, with no further developments, the VV contamination can be removed easily as shown in this work. Once the VV is removed, the composition of the vector containing material is similar to the other AAV vector systems and thus could be readily input into any of the existing methods of preparation of clinical-grade vectors.

In conclusion, the flexibility of vaccinia helper carrier allows this system to be readily adaptable and warrants further development and optimization to meet the requirement for human clinical trials.

## SUPPLEMENTARY DATA

Supplementary Data are available at NAR online: Supplementary Table 1 and Supplementary Figures 1–3.

## FUNDING

National Institutes of Health (NIH) [R01HL080789 and R01HL084381 to W.X.]; NIH under Ruth L. Kirschstein National Research Service Award T32-HL-007777 from the National Heart Lung and Blood Institute (to A.R.M.). NIAID intramural program (in part). Funding for open access charge: NIH.

*Conflict of interest statement*. None declared.

## Supplementary Material

Supplementary Data
