# Supplementary material for: A concept of eliminating nonhomologous recombination for scalable and safe AAV vector generation for human gene therapy
Source: Nucleic Acids Res. 2013 May 15;41(13):6609–17. doi: 10.1093/nar/gkt404 (PMC3711426; doi:10.1093/nar/gkt404)
Supplement: Supplementary Data [file supp_41_13_6609__index.html]

A concept of eliminating nonhomologous recombination for scalable and safe AAV vector generation for human gene therapy — A concept of eliminating nonhomologous recombination for scalable and safe AAV vector generation for human gene therapy — Supplementary Data 

# A concept of eliminating nonhomologous recombination for scalable and safe AAV vector generation for human gene therapy

## Supplementary Data

files

**Files in this Data Supplement:**

- Supplementary Data - pdf file
