# Supplementary material for: A concept of eliminating nonhomologous recombination for scalable and safe AAV vector generation for human gene therapy
Source: Nucleic Acids Res. 2013 May 15;41(13):6609–17. doi: 10.1093/nar/gkt404 (PMC3711426; doi:10.1093/nar/gkt404)
Supplement: Supplementary Data [file supp_gkt404_nar-00803-y-2013-File003.pdf]

Supplementary tables and figures

Supplementary Table 1: VV based AAV helpers can be amplified readily in BSC-1 cells and HeLa S3 cells. Each VV was used to infect the cells at MOI of 0.1 and collected at 48 hr postinfection. BSC-1 cells were cultured in 30 145mm tissue culture plates having around $3x10^8$ cells; HeLa S3 cells were cultured to $1.0x10^9$ in the spinner bottle.

Supplementary Figure 1 Stability of *rep* and *cap* genes in the recombinant vaccinia virus genomes. Vaccinia viruses harboring individual rep and cap genes were continuously propagated in BSC-1 cells for 10 times. For each VV at 10th generation, 8 single plaques were randomly picked up. The single plaque was amplified once in BSC-1 cells in 12-well plates and then used to infect BSC-1. Cell pellets were collected 48 hr postinfection. The expression of *cap* and *rep* genes was examined by western blot hybridization using anti-AAV capsid or Rep antibody, which was used to determine the genome stability of VV.

Supplementary Figure 2 Schematic representation of vaccinia carriers for expressing individual or combination of helper functions.

Supplementary Figure 3. Expression of canine factor VIII in Hemophilia A mouse model using AAV vectors produced by the novel system. Canine heavy chain (cHC) and light chain (cLC) were packaged in AAV serotype 8 either using Ad-AAV-CB-cHC, Ad-AAV-CB-cLC and vaccinia virus carriers (VV) or triple plasmid transfection (3plasmids). $4x10^{11}$ viral particles of AAV8-CB-cHC and $1x10^{11}$ viral particles of AAV8-CB-cLC were injected into HA mice via tail veins (n=3 for 3pds and n=4 for VV). Saline were injected as the negative control (n=3). Blood samples were collected via eye bleeding at 4 weeks after injection. The specific activity were calculated based on the aPTT assays. Factor VIII assay methods have been described previously in Chen L, et al. (2009). Enhanced factor VIII heavy chain for gene therapy of hemophilia A. Molecular therapy : the journal of the American Society of Gene Therapy 17: 417-424.

Supplementary Figure 1

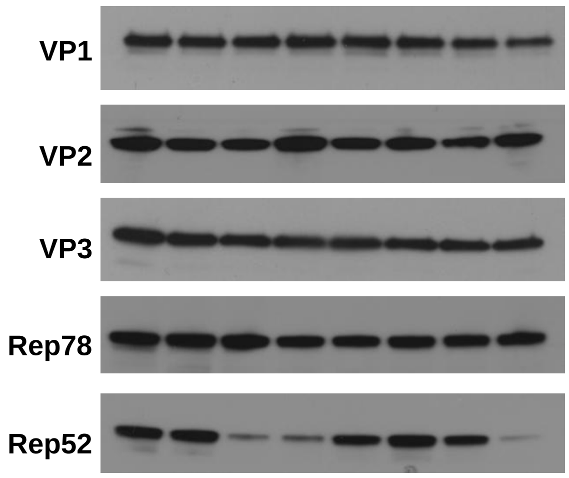

Supplementary Figure 2

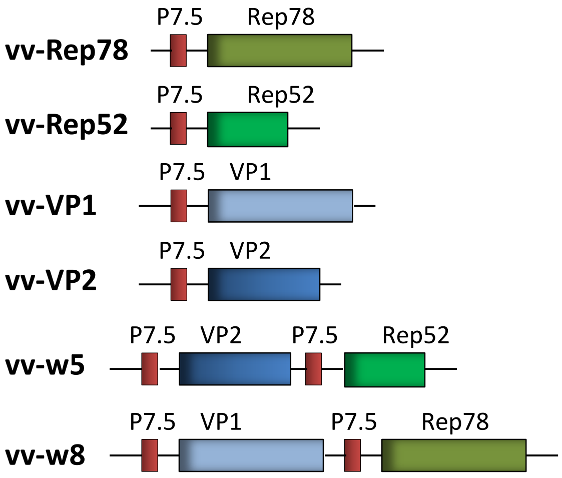

Supplementary Figure 3

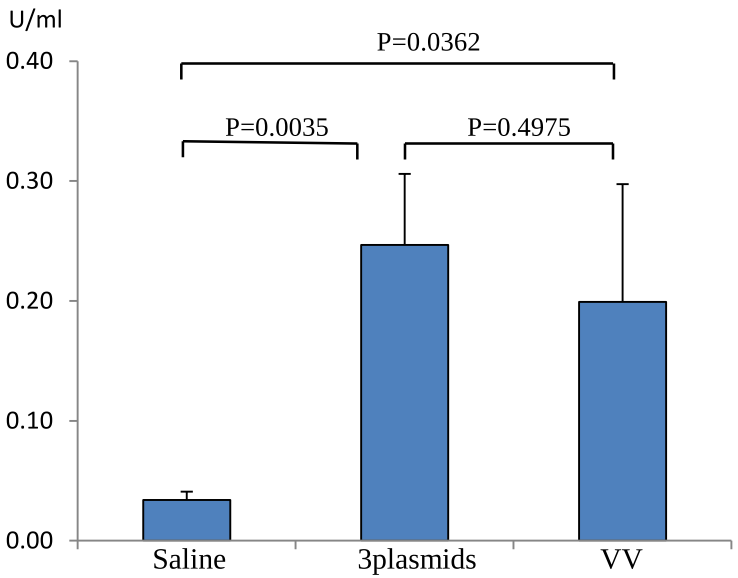

Supplementary Table 1

VV	BSC-1	HeLa S3
	Infectious units	Infectious units
VV-VP1	$4.2x10^{9}$	$2.1x10^{10}$
VV-VP2	$3.9x10^{9}$	$9.2x10^{9}$
VV-VP3	$3.7x10^{9}$	$8.2x10^{9}$
VV-Rep78	$1.6x10^{9}$	$4.7x10^{9}$
VVRep52	$1.8x10^{9}$	$4.6x10^{9}$
VV-w5	$2.5x10^{9}$	$1.3x10^{10}$
VV-w8	$2.4x10^{9}$	$9.6x10^{9}$
